# Plasma metabolites are altered before and after diagnosis of preeclampsia or fetal growth restriction

**DOI:** 10.1038/s41598-024-65947-9

**Published:** 2024-07-09

**Authors:** Lucy A. Bartho, Daniel R. McKeating, Susan P. Walker, Brunda Nijagal, Teresa M. MacDonald, Natasha Pritchard, Natalie J. Hannan, Anthony V. Perkins, Stephen Tong, Tu’uhevaha J. Kaitu’u-Lino

**Affiliations:** 1 Translational Obstetrics Group, The Department of Obstetrics and Gynaecology, Mercy Hospital for Women, University of Melbourne, 163 Studley Road, Heidelberg, VIC 3084 Australia; 2 Mercy Perinatal, Mercy Hospital for Women, Heidelberg, VIC Australia; 3 School of Medical Science, Griffith University, Gold Coast Campus, Parklands Drive, Southport, QLD 4215 Australia; 4 Metabolomics Australia, Bio21, University of Melbourne, Parkville, VIC Australia

**Keywords:** Translational research, Outcomes research

## Abstract

Metabolomics is the study of small molecules (metabolites), within cells, tissues and biofluids. Maternal metabolites can provide important insight into the health and development of both mother and fetus throughout pregnancy. This study assessed metabolic profiles in the maternal circulation prior to and at the time of diagnosis of preeclampsia and fetal growth restriction. Maternal plasma samples were collected from two independent cohorts: (1) Established disease cohort: 50 participants diagnosed with early-onset preeclampsia (< 34 weeks’ gestation), 14 with early-onset fetal growth restriction, and 25 gestation-matched controls. (2) Prospective cohort, collected at 36 weeks’ gestation before diagnosis: 17 participants later developed preeclampsia, 49 delivered infants with fetal growth restriction (birthweight < 5th centile), and 72 randomly selected controls. Metabolic evaluation was performed by Metabolomics Australia on the Agilent 6545 QTOF Mass Spectrometer. In the established disease cohort, 77 metabolites were altered in circulation from participants with preeclampsia – increased l-cysteine (3.73-fold), l-cystine (3.28-fold), l-acetylcarnitine (2.57-fold), and carnitine (1.53-fold) (p < 0.05). There were 53 metabolites dysregulated in participants who delivered a fetal growth restriction infant—including increased levulinic acid, citric acid (1.93-fold), and creatine (1.14-fold) (p < 0.05). In the prospective cohort, 30 metabolites were altered in participants who later developed preeclampsia at term – reduced glutaric acid (0.85-fold), porphobilinogen (0.77-fold) and amininohippuric acid (0.82-fold) (p < 0.05) was observed. There were 5 metabolites altered in participants who later delivered a fetal growth restriction infant – including reduced 3-methoxybenzenepropanoic acid (p < 0.05). Downstream pathway analysis revealed aminoacyl-tRNA biosynthesis to be most significantly altered in the established cohort in preeclampsia (13/48 hits, p < 0.001) and fetal growth restriction (7/48 hits, p < 0.001). The predictive cohort showed no significant pathway alterations. This study observed altered metabolites in maternal plasma collected before and after diagnosis of a preeclampsia or fetal growth restriction. While a significant number of metabolites were altered with established disease, few changes were observed in the predictive cohort. Thus, metabolites measured in this study may not be useful as predictors of preeclampsia or fetal growth restriction.

## Introduction

Preeclampsia and fetal growth restriction are major complications of pregnancy which can have lifelong health consequences^1^. Preeclampsia is characterised by new onset hypertension after 20 weeks' gestation, plus significant end-organ dysfunction (renal, hepatic, neurological abnormalities or fetal growth restriction) with or without proteinuria^2^. Broadly, preeclampsia can be classified as early-onset (delivery before 34 weeks’ gestation), or late-onset (delivery after 34 weeks' gestation)^3^.

Small-for-gestational-age (< 10th birthweight centile) fetuses are over-represented in pregnancies with poor perinatal outcomes. Many of these pregnancies will have placental insufficiency and fetal growth restriction. Fetal growth restriction describes a fetus that fails to achieve its genetic growth potential^4^. Fetal growth restriction and small-for-gestational-age fetuses are associated with an increased risk of stillbirth (3–fourfold)^5,6^. Despite their clinical significance, precise aetiology of preeclampsia and fetal growth restriction remain elusive, making early prediction and intervention difficult.

Metabolomics is the study of small molecules (metabolites), within cells, tissues and biofluids^7^. Metabolites represent the end products of cellular processes and provide an insight into an organism's metabolic status^8^. Maternal metabolites are important for optimal maternal health and fetal development throughout pregnancy^9^.

Metabolomics offers the ability to measure a range of metabolites in small sample volumes to identify subtle changes in the maternal circulation^7^. These alterations in metabolites may hold potential for: (1) predicting those at risk for developing preeclampsia or fetal growth restriction or, (2) understanding the metabolic pathways associated with each disease.

This study aims to assess metabolic profiles in maternal circulation prior to, and at the time of diagnosis of fetal growth restriction or preeclampsia.

## Results

### Maternal characteristics

The Established disease cohort includes blood samples from 50 early-onset preeclampsia, 14 fetal growth restriction, and 25 control pregnancies matched for gestational age from the Mercy Hospital for Women, Melbourne. Preeclampsia was defined by ACOG guidelines, and no subclinical infection was identified.

In the established disease cohort, significant differences were observed in maternal body mass index (BMI) (preeclampsia; p = 0.0004, FGR; p = 0.006), compared to controls (Table 1). No significant difference in gestation was observed for either collection, fetal sex, maternal age, parity, smoking status, or gestational diabetes status between the preeclampsia and fetal growth restriction cohorts, compared to gestation matched controls. As expected, birthweight was different in the fetal growth restriction cohort (p < 0.0001).

**Table 1:** Maternal characteristics and pregnancy outcomes for the Established disease cohort.

		Control (n = 25)	Preeclampsia (n = 50)	p value	Fetal growth restriction (n = 14)	p value
Gestation at collection (weeks) median (IQR)		28 [26–31]	30 [27–31]	0.288	27.5 [26.25–30.75]	0.937
Gestation at delivery (weeks), median (IQR)		39 [38–40]	30 [27–31]	< 0.0001	29 [27–37.5]	0.004
Birthweight (grams), median (IQR)		3480 [3180–3700]	1198.5 [839–1622.75]	< 0.0001	862.5 [634.25–2243.25]	< 0.0001
Fetal sex, no. (%)	Male	12 [48%]	21 [42%]	0.627	4 [28.6%]	0.248
	Female	13 [52%]	29 [58%]		10 [71.4%]	
Maternal age (years), median (IQR)		32.5 [28.75–34.25]	32 [29–34]	0.892	31 [30–35.25]	0.910
BMI (kg/m^2^), median (IQR)		24.2 [21.6–27.9]	28.5 [25.9–34.9]	0.0004	30.3 [25.6–37.1]	0.006
Parity no. (%)	0	10 [40%]	34 [68%]	0.110	11 [78.5%]	0.738
	1	10 [40%]	10 [20%]		2 [14.3%]	
	≥ 2	5 [20%]	6 [12%]		1 [7.2%]	
Smoking no. (%)	Non-smoker	20 [80%]	42 [84%]	0.871	10 [71.4%]	0.351
	Former smoker	5 [20%]	5 [10%]		3 [21.4%]	
	Smoker	0 [0%]	3 [6%]		1 [0%]	
GDM no. (%)	None	23 [92%]	43 [86%]	0.475	14 [100%]	0.272
	Diet-controlled	1 [4%]	4 [8%]		0 [0%]	
	Insulin-controlled	1 [4%]	3 [6%]		0 [0%]	

Data presented as median [25th–75th percentile] and as number (%) if categorical. Kruskal–Wallis tests were used for continuous data. Fisher’s exact tests were used for categorical variables. Fetal growth restriction is defined as an infant delivered < 5th birthweight centile. Significant difference is identified when p < 0.05.

*BMI* body mass index.

Next, plasma samples from the biomarker and ultrasound measures for preventable stillbirth cohort were collected at 36 weeks’ gestation. A case cohort of 144 samples were selected for metabolomics analysis, including 55 who later delivered infants with fetal growth restriction (birthweight < 5th centile), 22 women who later developed preeclampsia, and 72 randomly selected controls. Preeclampsia was defined according to the ACOG guidelines. At the time of collection, participants had no signs of pregnancy pathology (Table 2). In participants who developed preeclampsia at term, there were significant differences in birthweight (p = 0.006) and parity (p = 0.02). There were no differences in gestation at collection, crown-heel length, fetal sex, maternal age, BMI, smoking status, or gestational diabetes status.

**Table 2:** Maternal characteristics and pregnancy outcomes for the biomarker and ultrasound measures for preventable stillbirth (BUMPS) cohort.

		Control (n = 72)	Preeclampsia (n = 23)	p value	Fetal growth restriction (n = 55)	p value
Gestation at collection (weeks), median (IQR)		36.14 [35.64–36.42]	36.28 [35.53–36.57]	0.99	36.14 [35.71–36.42]	0.99
Birthweight (grams), median (IQR)		3570 [3266–3808]	3185 [2633–3458]	0.006	2615 [2443–2803]	< 0.0001
Fetal sex, no. (%)	Male	31 [43%]	11 [48%)	0.95	26 [46%]	0.71
	Female	41 [57%]	12 [52%]		29 [54%]	
Maternal age (years), median (IQR)		33 [31–35]	34 [31.75–37.25]	0.19	33 [31–36]	0.62
BMI (kg/m^2^), median (IQR)		24.60 [22.28–27.97]	28.21 [24.15–30.79]	0.06	25.34 [23.34–29.55]	0.61
Parity no. (%)	0	37 (51%)	19 (83%)	0.02	37 (69%)	0.12
	1	25 (35%)	4 (17%)		16 (28%)	
	≥ 2	10 (14%)	0 (0%)		2 (3%)	
Smoking no. (%)	Non-smoker	65 (90%)	21 (91%)	0.9	48 (87%)	0.5
	Former smoker	4 (6%)	1 (4%)		6 (11%)	
	Smoker	2 (3%)	1 (4%)		1 (2%)	
GDM no. (%)	None	63 (86%)	17 (74%)	0.09	50 (91%)	0.53
	Diet-controlled	8 (11%)	6 (26%)		3 (5%)	
	Insulin-controlled	2 (3%)	0 (0%)		2 (4%)	

Data presented as median [25th–75th percentile] and as number (%) if categorical. Kruskal–Wallis tests were used for continuous data. Fisher’s exact tests were used for categorical variables. Fetal growth restriction is defined as an infant birthed < 5th birthweight centile. Significant difference is identified when p < 0.05. BMI data missing for 1/73 control samples, and 1/55 fetal growth restriction samples. Birth weight missing for 1/55 fetal growth restriction samples.

*BMI* body mass index.

In participants who delivered an infant that was growth restricted at term, there were significant differences in birthweight (p < 0.0001) and crown-heel length (p < 0.0001). There were no differences observed for either gestation at collection, fetal sex, maternal age, BMI, parity, smoking status, and gestational diabetes status.

### Exploratory metabolomic analysis: established disease cohort

**Figure 1:**
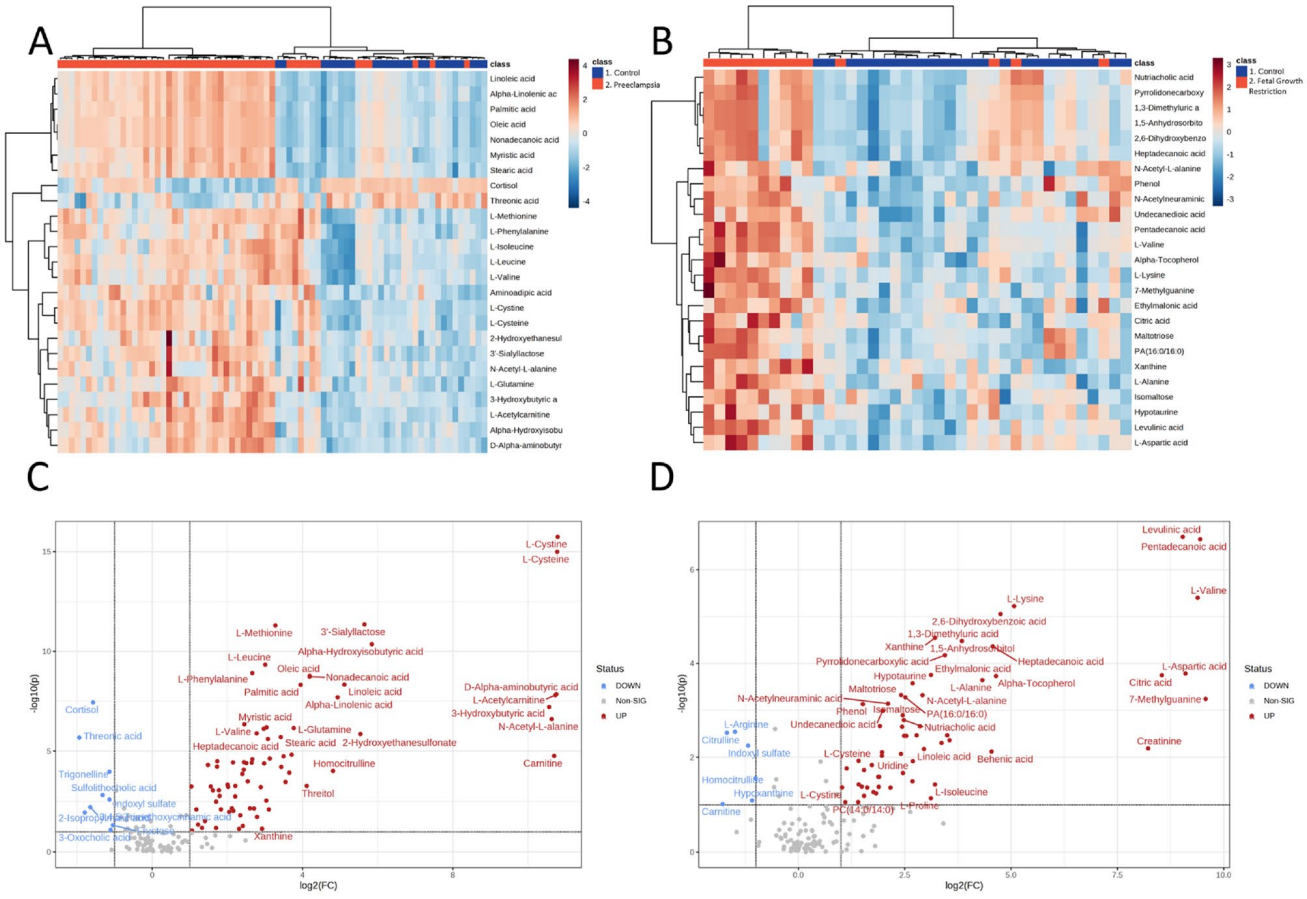
Hierarchical cluster analysis via heatmaps and volcano plots revealed metabolite profile differences in the metabolome of plasma collected at < 34 weeks’ gestation from pregnancies complicated by early-onset preeclampsia (**A** and **C**) and fetal growth restriction (**B** and **D**). (**A**) Heatmap and dendrogram using Euclidean distance measurement and ward clustering method of top 25 significant metabolites for control (Blue), and preeclampsia (Red) pregnancies. (**B**) Heatmap and dendrogram using Euclidean distance measurement and ward clustering method of top 25 significant metabolites for control (Blue), and preeclampsia (Red) samples collected from Established disease cohort. Colours for samples are denoted in the top x-axis of both (**A**) and (**B**). All metabolites have been scaled. Heatmap scales are denoted by red being higher, blue being lower, with white residing in the middle; this scale is used for each of the metabolites listed on the y-axis. Ward clustering algorithm used for dendrograms. (**C**) Established fetal growth restriction volcano plot, x-axis indicates the fold change (FC) in Log2 format, whilst the y-axis is t-test significance in − Log10. (**D**) Established fetal growth restriction volcano plot, x-axis indicates the fold change (FC) in Log2 format, whilst the y-axis is t-test significance in − Log10. In both instances of (**C**) and (**D**) standard *t*-test was conducted for p value calculations, significance was considered at p value of 0.1 along with a FC of 2.0. Ratios of Fold changes are calculated as disease/ control with blue indicating decreased fold change and red indicating an increased fold change.

The established disease cohort study successfully identified 174 metabolites for analysis with Level 1 identification (metabolomics standard initiative^10^) based on the matching to 550 authentic standards in the MA library. Relative to controls, 95 metabolites were found to be significantly altered in the maternal circulation from participants with preeclampsia, and 80 dysregulated in participants who delivered a fetal growth restricted infant. Heatmaps were used to identify metabolite changes in maternal plasma between preeclampsia (Fig. 1A) and fetal growth restriction pregnancies, (Fig. 1B) compared to gestation-matched controls. Figure 1A indicates clear groupings between samples collected from participants with diagnosed preeclampsia (delivered < 34 weeks’ gestation), compared to gestation-matched controls. Figure 1B also indicates a clear separation between participants who delivered a growth restricted infant, compared to gestation-matched controls. This demonstrates that at the time of diagnosis, of either preeclampsia or fetal growth restriction, there are marked differences in the metabolic profiles within the maternal circulation.

Volcano plots demonstrate the identified metabolites that significantly differ between groups. Figure 1C demonstrates metabolites found to be significantly altered in participants with preeclampsia, compared to gestation matched controls. Overall, participants diagnosed with preeclampsia had a higher concentration of most altered metabolites. The top five changes in metabolites based on fold changes were L-cystine, l-cysteine, l-acetylcarnitine, carnitine, and d-Alpha-aminobutyric acid, compared to controls (p < 0.05, Supplementary Table 1). Participants who delivered growth restricted infants relative to controls, The top 5 metabolite changes were levulinic acid, pentadecanoic acid, l-valine, l-aspartic acid, citric acid, and creatine, compared to controls (p < 0.05, Supplementary Table 2).

### Exploratory metabolomic analysis—predictive cohort: BUMPS

**Figure 2:**
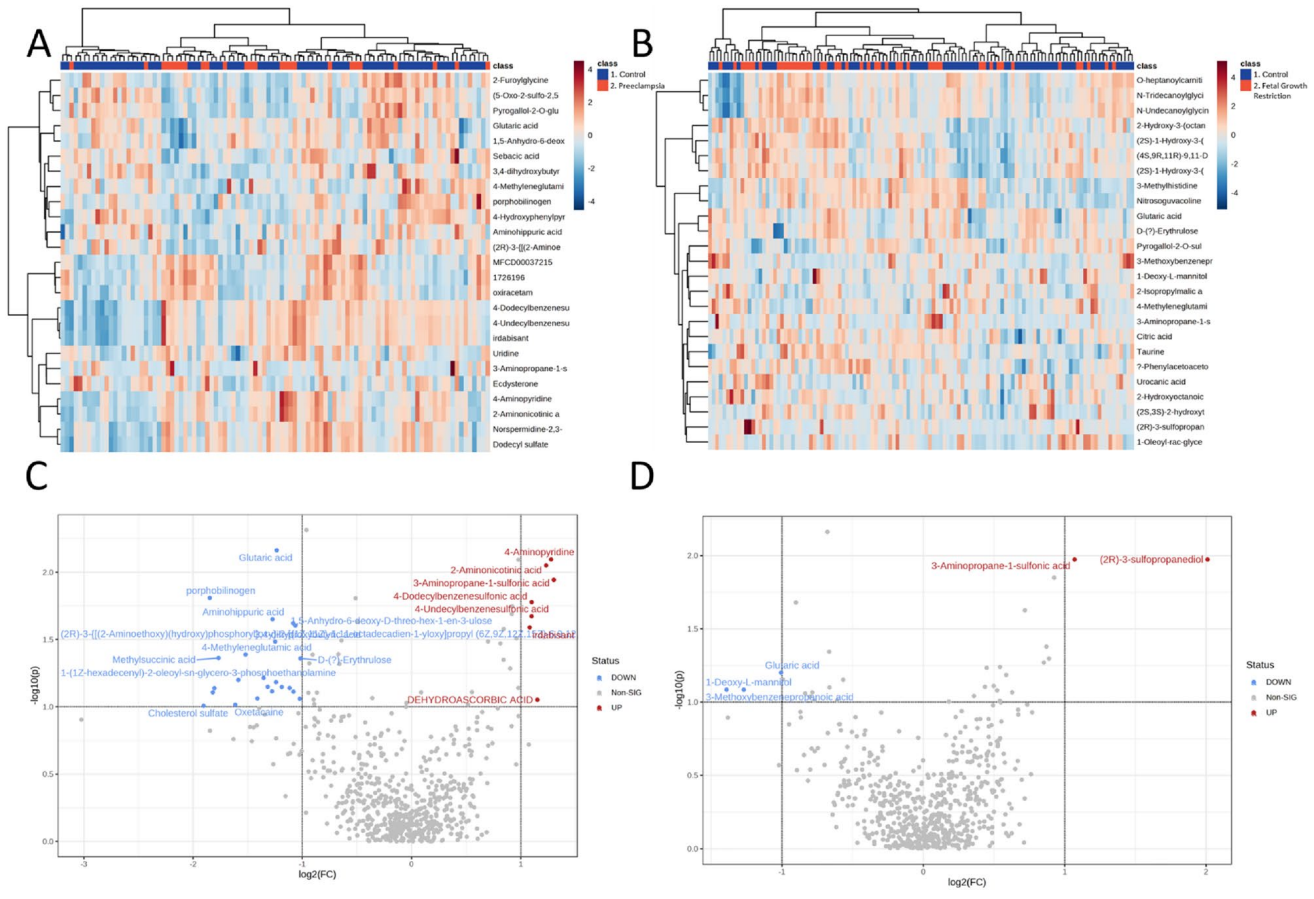
Heatmaps and volcano plots of metabolite data collected from the Biomarker and Ultrasound Measures for Preventable Stillbirth (BUMPS). Heatmaps, dendrograms and volcano plots of maternal plasma collected at 36 weeks’ gestation prior to diagnosis of term preeclampsia (**A** and **C**) or delivered a fetal growth restricted infant (**B** and **D**), compared to controls. (**A**) Heatmap and dendrogram using Euclidean distance measurement and ward clustering method of top 25 significant Metabolites for control (Blue), and preeclampsia (Red) pregnancies. (**B**) Heatmap and dendrogram using Euclidean distance measurement and ward clustering method of top 25 significant Metabolites for control (Blue), and preeclampsia (Red) samples collected from BUMPs cohort. Colours for samples are denoted in the top x-axis of both (**A**) and (**B**). All metabolites have been scaled. Heatmap scales are denoted by red being higher, blue being lower, with white residing in the middle; this scale is used for each of the metabolites listed on the y-axis. Ward clustering algorithm used for dendrograms. (**C**) Established fetal growth restriction volcano plot, x-axis indicates the fold change (FC) in Log2 format, whilst the y-axis is *t*-test significance in − Log10. (**D**) BUMPS fetal growth restriction volcano plot, x-axis indicates the fold change (FC) in Log2 format, whilst the y-axis is t-test significance in − Log10. In both instances of (**C**) and (**D**) standard t-test was conducted for p value calculations, significance was considered at p value of 0.1 along with a FC of 2.0. Ratios of Fold changes are calculated as disease/ control with blue indicating decreased fold change and red indicating an increased fold change.

Metabolomic analysis of the BUMPS samples successfully detected 631 molecular features in an untargeted matrix, consisting of 143 identified (matched to the MA authentic standard library run with the batches). Whilst similar numbers of metabolites were identified across the two project batches, an additional 488 metabolites were putatively annotated (following stringent curation of the Acquire X mass spectral peaks) in the BUMPS samples permitted by the high resolution and mass accuracy afforded by the Orbitrap MS. In total, there were 30 metabolites significantly altered in participants who developed preeclampsia and 5 in participants who later delivered a growth restricted infant. Figure 2A indicates no clear groupings between participants who were later diagnosed with term preeclampsia, compared to gestation-matched controls. Figure 2B identifies no clear separation between fetal growth restriction (delivered at term), compared to gestation-matched controls. This suggests that prior to disease onset, metabolic profiles within the maternal circulation are not dramatically altered compared to those who have received a disease diagnosis. Essentially, there are changes in profiles between Established and BUMPS cohort, although the changes were more severe in the Established cohort.

Volcano plots present metabolites identified as significantly altered between groups. Figure 2C identifies metabolites significantly altered in participants who later developed preeclampsia at term, compared with gestation matched controls. Overall, participants who were later diagnosed with preeclampsia had a lower relative level of metabolites glutaric acid, porphobilinogen, amininohippuric acid and cholesterol sulfate, compared to gestation matched controls (top fivefold changes, Supplementary Table 3). Participants who delivered growth restricted infants at term had lower relative level of metabolites, including glutaric acid, 1-Deoxy-l-mannitol and 3-Methoxybenzenepropanoic acid (Supplementary Table 4).

**Figure 3:**
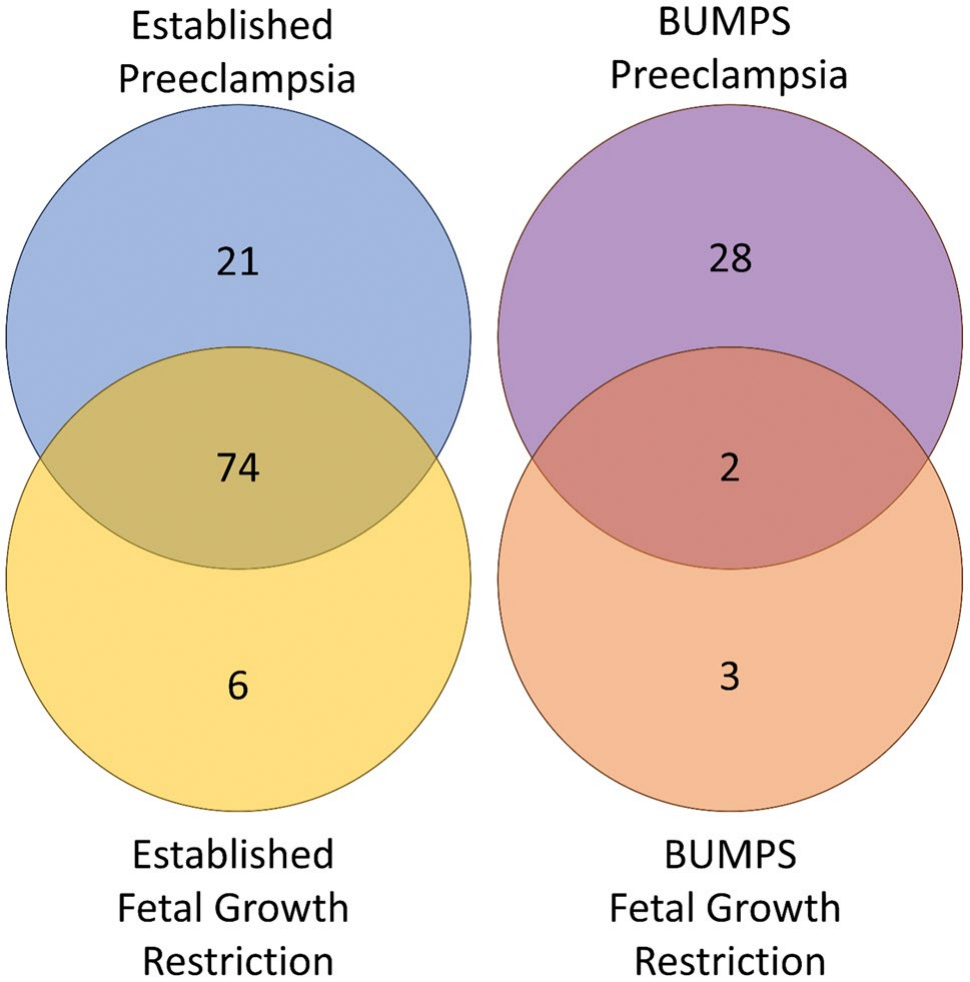
Venn diagram of the metabolite cross over between established preeclampsia (Blue), Established fetal growth restriction (Yellow), BUMPS preeclampsia (Purple), and BUMPS fetal growth restriction (orange). Numbers indicate the number of significantly different metabolites that are exclusive to that cohort and outcome. Where circles overlap, indicates the number of metabolites that are significantly different from controls that are shared between outcomes and/or cohort.

We assessed whether there was any cross over in metabolites dysregulated with a pregnancy pathology in the established cohort and the BUMPs cohorts (Fig. 3). In the established disease cohort, there were a total of 101 dysregulated metabolites. There were 21 metabolites significantly altered with preterm preeclampsia, 6 altered in participants who delivered with preterm fetal growth restriction, and 74 altered metabolites common to both pathologies. In the BUMPs cohort, there were a total of 33 dysregulated metabolites. There were 28 metabolites significantly altered in those who later developed preeclampsia, 3 altered with participants who delivered a fetal growth restricted infant at term, and 2 altered metabolites common to both pregnancy pathologies (3-Aminopropane-1-sulfonic acid and Glutaric acid) (Supplementary Tables 3 and 4) (Table 3).

**Table 3:** Top affected pathways in the maternal circulation both preeclampsia and fetal growth restriction.

Pathway	Total	Preeclampsia (established)				Fetal growth restriction (established)				Preeclampsia (BUMPS)			
		Hits	Raw p	− log10	Holm adjust	Hits	Raw p	− log10	Holm adjust	Hits	Raw p	− log10	Holm adjust
Aminoacyl-tRNA biosynthesis	48	13	3.9E-12	11.41	3.28E-10	7	4.1E-6	5.39	0.0003				
Arginine biosynthesis	14	4	0.0002	3.73	0.01	3	0.001	2.97	0.08				
d-Glutamine and d-glutamate metabolism	6	3	0.0002	3.70	0.01	3	6.3E-5	4.19	0.005				
Valine, leucine and isoleucine biosynthesis	8	3	0.0005	3.26	0.04	3	0.0002	3.75	0.01				
Biosynthesis of unsaturated fatty acids	36	5	0.001	3.002	0.08	5	0.0001	3.80	0.01				
Phenylalanine, tyrosine and tryptophan biosynthesis	4	2	0.003	2.54	0.23								
Alanine, aspartate and glutamate metabolism	28	4	0.004	2.52	0.24	3	0.008	2.08	0.63				
Butanoate metabolism	15	3	0.004	2.40	0.31	3	0.001	2.88	0.10				
Pantothenate and CoA biosynthesis	19	3	0.008	2.10	0.61	3	0.003	2.57	0.21				
Ubiquinone and other terpenoid-quinone biosynthesis	9	2	0.02	1.79	1								
Porphyrin and chlorophyll metabolism	30	2	0.14	0.84	1	1	0.38	0.42	1	1	0.11	0.95	1
Arginine and proline metabolism	38	2	0.21	0.68	1	1	0.45	0.34	1	1	0.14	0.85	1
Histidine metabolism	16	1	0.31	0.52	1	1	0.22	0.65	1	1	0.06	1.21	1
Steroid hormone biosynthesis	85	1	0.86	0.06	1					1	0.28	0.54	1
Glycosylphosphatidylinositol (GPI)-anchor biosynthesis	14									1	0.05	1.27	1

Fetal growth restriction cases from the BUMPs cohort were excluded from this table due to lack of significance within the same altered pathways. Total indicates the number of metabolites within a given pathway. Hits indicates the number of metabolites altered within that pathway. Analysis conducted via MetaboAnalyst 5.0 pathway analysis, Hypergeometric test used for enrichment, topology analysis by relative-betweenness centrality, using KEGG pathway library.

### Pathway analysis

The top metabolic pathways in each cohort were further investigated using downstream pathway analysis software (MetaboAnalyst). The top pathways altered in the established disease cohort with either pregnancy pathology (preeclampsia or fetal growth restriction) was Aminoacyl-tRNA biosynthesis (13/48 metabolites altered); arginine biosynthesis (4/14 metabolites altered); d-glutamine and d-glutamate metabolism (3/6 metabolites altered); valine, leucine and isoleucine biosynthesis (3/8 metabolites altered) and biosynthesis of unsaturated fatty acids (5/36 metabolites altered). There were no significantly altered pathways observed preceding the diagnosis of pregnancy pathologies in the BUMPs cohort.

## Discussion

The aim of this study was to assess metabolic profiles in maternal circulation before and after diagnosis of a pregnancy pathology (preeclampsia or fetal growth restriction). We performed metabolomics analyses using LC–MS on two independent cohorts, collected from two different timepoints in pregnancy. The first, established disease, is a tissue bank analysis collected at the time of disease diagnosis. These samples are from a nested case–control study of participants with preterm diagnoses (< 34 weeks’ gestation) compared to gestation-matched controls. This is different to the predictive cohort which used a case-cohort study design. This design is advantageous as it allows for randomly selected controls to be used in both disease outcomes^11^.

In the established disease cohort, we found distinct alterations in metabolic profiles in maternal plasma with a pregnancy pathology (preeclampsia or fetal growth restriction), compared to gestation matched controls. Participants with early-onset preeclampsia had a higher abundance of l-cysteine, l-cystine, l-acetylcarnitine and carnitine. l-cysteine is a semi essential amino acid and a protein thiol – an organic compound that contains a thiol group (-SH; a sulphur and hydrogen atom attached to a carbon atom). In the blood, l-cysteine is rapidly oxidised to its disulfide form, l-cystine under normoxic conditions^12^. However, inside cells, l-cysteine is the predominant form due the intracellular environment favouring the reduced form^13^. Studies show an imbalance of extracellular cysteine/cystine may be reflective of proinflammatory redox signalling in cardiovascular disease and a key indicator of vascular health^14,15^. These changes are observed in response to oxidative stress, which is a key characteristic of preeclampsia^14,15^. Further, work from Raijmakers et al. corroborates our findings as they found increased free and oxidised thiols, cysteine and homocysteine, in whole blood in participants with preeclampsia at 32 weeks’ gestation^16^.

Carnitine and acylcarnitines play a key role in ß-oxidation. They are responsible for transporting acyl-groups (i.e. fatty acids) from the cytoplasm to the mitochondria to produce energy^17^. A study investigating the insulin resistance in muscle tissue^18^ found incomplete fatty acid ß-oxidation in muscle tissue causes acylcarnitine-induced oxidative stress contributing to insulin resistance^18^. Our study supports a study by Thiele et al. who found a significant increase in short- and long-chain acylcarnitines in plasma collected from women with preeclampsia, compared to normotensive controls^19^.

Participants with preterm fetal growth restriction had a higher abundance of levulinic acid, citric acid, and creatine. Levulinic acid is a secondary metabolite – which are non-essential metabolites that may play a role in molecular signalling within the body. A study by Sulek et al. investigated the metabolic profile in maternal hair for predictive biomarkers for fetal growth restriction^20^. They identified a multivariate predictive model combining 5 metabolites to discriminate between fetal growth restriction and controls. One of these was levulinate, the salt of levulinic acid (AUC 0.998, < 10th birth centile)^20^. Although, this study identified this biomarker earlier than our study, future work should focus on validating our finding in an independent cohort before the diagnosis of preterm fetal growth restriction.

To further understand altered metabolites and their pathways, our datasets were analysed using the KEGG pathway analyser. In our established disease cohort, metabolites associated with the aminoacyl-tRNA biosynthesis pathway were significantly upregulated in both preeclampsia and fetal growth restriction groups. This finding is similar to Gao et al.^21^, where the aminoacyl-tRNA biosynthesis pathway was upregulated in gastric cancer tissues compared to noncancerous tissues. In our study, we observed a significant elevation in metabolites associated with this pathway in plasma collected from fetal growth restriction and preeclampsia pregnancies, compared to controls. The dysregulation of aminoacyl-tRNA biosynthesis may impact cellular functions like oxidative stress, a known contributor to preeclampsia and fetal growth restriction^22^. More research is required to confirm this hypothesis.

In our prospective cohort (BUMPS), plasma samples were collected at 36 weeks’ gestation before diagnosis of a pregnancy pathology (preeclampsia or fetal growth restriction). In this analysis, we identified a small number of metabolites that were significantly dysregulated in participants who later developed a pregnancy pathology at term. Interestingly, the metabolites from this cohort were significantly reduced in plasma from women who later developed term preeclampsia or fetal growth restriction. Participants diagnosed with preeclampsia at term had a lower abundance of metabolites, like glutaric acid, porphobilinogen, amininohippuric acid and cholesterol sulfate, compared to gestation matched controls. Those who delivered infants with fetal growth restriction had relatively lower levels of metabolites, glutaric acid and 3-Methoxybenzenepropanoic acid. None of these metabolites had strong predictive capabilities, with none reaching higher than an area under the curve (AUC) of 0.67 (Supplementary Table 5). There is limited research on these metabolites in pregnancy, future research should investigate these metabolites and their potential role in preeclampsia and fetal growth restriction. A study by Sovio, U. et al. explored the use of maternal serum metabolite ratios for identifying term growth restriction^6^. Notably, their significant markers—1-(1-enyl-stearoyl)-2-oleoyl-GPC (P-18:0/18:1), 1,5-anhydroglucitol, 5α-androstan-3α,17α-diol disulfate, and N1,N12-diacetylspermine—were absent in our sample analysis^6^. This discrepancy may stem from differing methodologies: QToF-MS was used in our study, where Sovio et al. employed ultrahigh performance liquid chromatography-tandem mass spectrometry (UPLC-MS/MS). Despite analysing similar sample sets, these methodological variances highlight the lack of standardised procedures and methodologies across different institutions as well as countries. For metabolomics to be used effectively within a clinical environment, targeted methodologies would be required to provide the best possibility of translating biomarkers between institutes.

A comprehensive systematic literature review of metabolomics in PE diagnosis conducted by Nobakht M. Gh, B. F. highlighted patients with PE had altered levels of lipids, amino acids, inflammation, oxidative stress, coagulation, and angiogenesis metabolites^23^. Within the established cohort we noticed similar changes such as aminoacyl-tRNA-biosynthesis, along with arginine biosynthesis, which was highlighted as a possible marker. Arginine biosynthesis produces vasodilatory effects by elevating nitric oxide production through the Arg-NO pathway, reducing blood pressure. However, this change was not exclusive to PE in the established cohort, as we found significant alterations in patients with FGR. Therefore, alterations in arginine biosynthesis may provide an insight into pregnancy health.

Similarly, a research paper on non-targeted metabolomic study of fetal growth restriction by Chen, F. et al. found abnormal changes in amino acid metabolism in amniotic fluid samples. Although they had a low number of samples (n = 28), these results were similar to the results found in the established cohort, but not BUMPS cohort^24^. The changes may be associated with early onset fetal growth restriction, but not late onset.

In the BUMPs study, there were no significant changes in metabolic pathways to report. This may be because at collection of each sample, the participants were clinically healthy and had no signs of disease development. Thus, metabolites measured at 36 weeks’ gestation alone may not serve as predictive biomarkers but could be combined with gene and/or protein biomarkers.

The overlap of control samples in Fig. 1A,B and Fig. 2A,B clustering is likely due to the similarity of metabolites within those participants to the complication groups. The overlap is expected and, in a sense, if these metabolites were used to construct any models of prediction, then these would be classified as false positives. The inverse is also true, any complicated samples that are clustered with control samples are likely to be false negatives. Though many samples in the Established cohort are separated there is an overlap of mainly false negatives, however the BUMPS cohort the distinction between groups is almost negligible, and as a result seems to indicate that the earlier an onset of PE or FGR, the more likely there is a systemic metabolomic shift in maternal metabolism.

In this study, the established and BUMPs cohorts were run on the LC–MS at different times in pregnancy. We note that although these samples were run on the machine at different timepoints, we do not directly compare metabolites across cohorts. Therefore, we were unable to confidently make direct comparisons of data. In metabolomics studies, samples need to be collected in a consistent manner for optimal performance^25^. Our study performed appropriate collection procedures – upon collection, samples were immediately spun down and snap frozen. Unfortunately, due to the design of the study, we were unable to eliminate population differences.

Moreover, our study sought to compare two cohorts: an established disease cohort—diagnosed at the time of collection, whilst BUMPs was a prospective and predictive cohort. This led to divergent findings between the two cohorts, limiting our capacity for intricate analysis, as well as assessing the profiles of metabolites in circulation prior to and at the time of diagnosis of preeclampsia or fetal growth restriction. Subsequent studies could consider pooling samples from cohorts with matched samples across gestation to enable a fair comparison of metabolomic profiles, or alternatively, adhere to uniform criteria for sample collection.

This study investigated the metabolic profile of two independent cohorts, from pregnant women before and after development of preeclampsia and fetal growth restriction. The metabolic profiles observed in the Established disease cohort (Established) and the Predictive cohort (BUMPS) underpin the changes between these conditions at different times of pregnancy. The significant metabolic differences in early-onset preeclampsia further emphasises the need for in-depth exploration of these metabolic pathways. These findings indicate the need for further research and a deeper understanding of metabolic changes associated with preeclampsia and fetal growth restriction, with the aim of improving early detection and intervention strategies.

## Methods

### Established disease cohort (established)

The established disease cohort is a collection of samples obtained from the Mercy Hospital for Women, Melbourne, Australia. Blood samples were collected from singleton pregnancies consisting of 50 participants with established early-onset preeclampsia (< 34 weeks’ gestation), 14 participants with diagnosed fetal growth restriction (birthweight < 5th centile and no preeclampsia), and 25 gestation-matched controls with no evidence of placental insufficiency. For all cohorts, preeclampsia was defined according to the American College of Obstetricians and Gynaecologists (ACOG) guidelines^26^. Ethics approval was granted by Mercy Health Human Research Ethics Committee (R11/34). Participants presenting to the Mercy Hospital for Women gave informed, written consent for sample collection. No evidence of subclinical infection was identified. Clinical characteristics have been provided in Table 1. Whole blood was collected in a 9 mL ethylenediaminetetraacetic acid tube, samples were centrifuged at 1000×*g* for 10 min, and plasma was stored at − 80 °C until further analysis.

### Predictive pregnancy complication cohort (BUMPS)

The Biomarker and Ultrasound Measures for Preventable Stillbirth (BUMPS) study is a large prospective study conducted at the Mercy Hospital for Women, Melbourne, Australia. This study involved the collection of blood at 36 weeks’ gestation (35^0+^–37^0+^) prior to diagnosis of a pregnancy disorder. A case cohort of 144 samples were selected for metabolomics analysis, including 55 who later delivered infants with fetal growth restriction (birthweight < 5th centile), 22 women who later developed preeclampsia, and 72 randomly selected controls. Preeclampsia was defined according to the ACOG guidelines^26^. Ethical approval was obtained from the Mercy Health and Human Research Ethics Committee (Approval number: 2019–012) and participants gave informed, written consent. Clinical characteristics have been provided in Table 2. Whole blood was collected in 9 mL EDTA tubes, centrifuged at 1000xg for 10 min, and plasma was stored at − 80 °C until further analysis.

### Metabolomic analyses

Metabolomics analyses were conducted by Metabolomics Australia, Bio21 Molecular Science and Biotechnology Institute, University of Melbourne, Parkville, Australia. All experiments were performed in accordance with relevant guidelines and regulations. The methodology conducted was adapted from their previous publication^27^. Briefly, 20uL of sample was aliquoted for LC–MS analysis. An additional 20 ul aliquot of each sample were pooled to create a plasma quality control (PQC), from which aliquots were taken for extraction with the samples. The plasma sample and PQC aliquots were extracted in a solution of 180ul of Acetonitrile/Methanol (1:1 v/v) spiked with 2 μM 13C-sorbitol, 2 μM 13C15N-AMP, and 2 μM 13C15NUMP for analysis as internal standards.

Metabolite separation was performed on a ZIC^®^-pHILIC column (5 μm particle size, 150 Å ~ 4.6 mm, Merck SeQuant^®^) maintained at 25 °C, 7uL of sample was injected onto an Agilent 1260 (Santa Clara, CA, USA) HPLC using with mobile phases 20 mM ammonium carbonate (pH 9.0; Sigma-Aldrich; Solvent A) and 100% acetonitrile (solvent B) at a flow rate of 300 μL/min. The gradient began at a composition of 80% solvent B and solvent B was decreased to 50% at 15.5 min, 30% at 17.5 and 5% at 18.5-min before returning to 80% at 23 min for 10 min. For the established disease cohort, mass spectrometry analysis was performed on an Agilent 6545 series quadrupole time-of-flight mass spectrometer (QTOF MS) (Agilent Technologies, (Santa Clara, CA, USA), where metabolites were negatively ionized. Detailed Q-Tof MS parameters can be found in the publication (11).

Samples were randomised, with QC’s every 5 samples. To monitor batch preparation effects and instrument performance, PBCs were run every 10 samples. For the BUMP cohort, metabolite separation was performed on Vanquish Horizon UHPLC system (Thermo Scientific) with chromatography conditions as described previously. Metabolite detection was performed on a Orbitrap ID-X Tribrid Mass Spectrometer (Thermo Scientific) coupled to heated electrospray ionisation (H-ESI) source with the following conditions: sheath gas flow 40 arbitrary units (Arb), auxiliary gas flow 10 Arb, sweep gas flow 1 Arb, ion transfer tube temperature 275 °C, and vaporizer temperature 320 °C. The RF lens value was 35%. Data was acquired in positive polarity with spray voltages of 3500 V.

AcquireX workflow was used with a pooled-reference QC and a blank sample to aid in metabolite identification. MS1 scans and MS2 spectra were collected on [M + H]– ions in negative polarity over six runs by using iterative DDA. A stepped HCD Collision Energies of 20, 35 and 50% were used as the collision energy.

A further pooled biological quality control (pbQC), achieved by pooling 10ul aliquot of each extract, were run every 10 samples. Consequently, a QC sample was run every 5 samples. The metabolomics batches were assessed prior to data analysis to ensure a majority of the variability in the areas of the ISTDs in the samples, pbQC and pQCs were less than 25% coefficient of variation (% CV) and the retention times were < 2% CV. Batch correction was not performed between the two metabolomics batches. Instead, the batches were utilised independently for the relative comparison of the control patient samples to the disease onset patients.

### Metabolite identification

For the established disease cohort, relative abundances based on area under the metabolite peak were obtained on MassHunter Quantitative Analysis B.07.00 (Agilent Technologies). After the removal of low quality and unreliably detected peaks, 174 metabolites were detected in the established disease cohort (< 34 weeks) with Level 1 identification (metabolomics standard initiative^10^) based on the retention time and molecular masses matching (error < 10 ppm) to 550 authentic standards in the Metabolomics Australia in-house library which were run with the two projects.

The BUMPS cohort data processing (peak detection, peak alignment, and peak integration) was performed in Compound Discoverer 3.1 software (Thermo Fisher Scientific). After data clean-up, 143 metabolites (Level 1) were identified from the MA library and a further 488 putatively annotated via the Acquire X mass spectral peaks, which were searched against both ChemSpider™ chemical structure database (2 ppm mass tolerance) and mzCloud spectral library (precursor and fragment mass tolerance, 5 ppm). Three data sources were selected via the ChemSpider database: Human Metabolome Database (HMDB), Kyoto Encyclopaedia of Genes and Genomes (KEGG) and Biocyc.

### Statistical analysis

Initial data analyses were performed using GraphPad Prism v.9.5.0 (GraphPad Software, LLC). Maternal characteristics were compared for women diagnosed with early-onset preeclampsia (established disease cohort), or prior to diagnosis (BUMPS cohort) compared to normotensive, gestation-matched controls, per cohort. Variables were initially examined for normality, outliers, and extreme values by means of Shapiro–Wilk’s, and Kolmogorov-Sminov’s tests using a Mann–Whitney *U* test for continuous data and Fisher’s exact test for categorical data.

For the metabolomics, both cohorts were analysed independently using MetaboAnalyst 5.0 (https://www.metaboanalyst.ca/). Metabolites were log transformed, mean centred and normalised by the standard deviation to produce a set of pre-processed metabolite measurements for further analysis.

Heatmaps coupled with dendrograms were produced to visualise sample clusters illustrating the similarity or dissimilarity between samples. Top differentially expressed metabolites were presented on each heatmap. Dendrograms were constructed using Euclidean distance measurement and ward clustering method. Volcano plot analyses were conducted to visualise significant differences between groups.

To determine significantly dysregulated metabolites, the p value for the coefficient representing the metabolite was calculated with p < 0.05 used as a cut off for significance. Pathway analysis was conducted in MetaboAnalyst using the HMDB ID of each metabolite, Hypergeometric test used for enrichment, topology analysis by relative-betweenness centrality, using KEGG pathway library.

### Supplementary Information

Supplementary Tables.

## Data Availability

Data available upon request. Contact Lucy Bartho for data information.
