# Supplementary material for: Plasma metabolites are altered before and after diagnosis of preeclampsia or fetal growth restriction
Source: Sci Rep. 2024 Jul 9;14:15829. doi: 10.1038/s41598-024-65947-9 (PMC11233654; doi:10.1038/s41598-024-65947-9)
Supplement: Supplementary file 1 — Supplementary Tables. [file 41598_2024_65947_MOESM1_ESM.docx]

**Supplementary table 1: Significant differences of metabolites in plasma collected from Established disease cohort – Participants diagnosed with preeclampsia, compared to gestation matched controls.**

|  | FC | log2(FC) | P value | -LOG10(p) | Q Value | -LOG10(p) |
| --- | --- | --- | --- | --- | --- | --- |
| L-Cystine | 1771.4 | 10.791 | 1.79E-16 | 15.747 | 2.90E-14 | 13.537 |
| L-Cysteine | 1755.1 | 10.777 | 1.01E-15 | 14.997 | 8.15E-14 | 13.089 |
| 3'-Sialyllactose | 50.129 | 5.6476 | 4.47E-12 | 11.349 | 2.02E-10 | 9.6938 |
| L-Methionine | 9.6986 | 3.2778 | 5.00E-12 | 11.301 | 2.02E-10 | 9.6938 |
| Alpha-Hydroxyisobutyric acid | 57.412 | 5.8433 | 4.38E-11 | 10.358 | 1.42E-09 | 8.8477 |
| L-Leucine | 8.0385 | 3.0069 | 4.61E-10 | 9.3363 | 1.24E-08 | 7.9049 |
| L-Phenylalanine | 6.3337 | 2.663 | 1.23E-09 | 8.9097 | 2.85E-08 | 7.5453 |
| Oleic acid | 18.268 | 4.1913 | 1.70E-09 | 8.7685 | 3.34E-08 | 7.4756 |
| Nonadecanoic acid | 18.254 | 4.1902 | 1.86E-09 | 8.7309 | 3.34E-08 | 7.4756 |
| Linoleic acid | 34.631 | 5.114 | 4.66E-09 | 8.3318 | 7.07E-08 | 7.1506 |
| Palmitic acid | 15.441 | 3.9487 | 4.80E-09 | 8.3188 | 7.07E-08 | 7.1506 |
| D-Alpha-aminobutyric acid | 1727.6 | 10.755 | 1.42E-08 | 7.8484 | 1.91E-07 | 6.7181 |
| L-Acetylcarnitine | 1696.6 | 10.728 | 1.57E-08 | 7.8041 | 1.96E-07 | 6.7085 |
| Alpha-Linolenic acid | 30.558 | 4.9335 | 2.03E-08 | 7.6927 | 2.35E-07 | 6.6293 |
| Cortisol | 0.33589 | -1.5739 | 3.62E-08 | 7.4413 | 3.91E-07 | 6.4078 |
| 3-Hydroxybutyric acid | 1511.8 | 10.562 | 6.18E-08 | 7.209 | 6.26E-07 | 6.2036 |
| N-Acetyl-L-alanine | 1585.5 | 10.631 | 2.52E-07 | 6.5989 | 2.40E-06 | 5.6198 |
| L-Valine | 5.4654 | 2.4503 | 4.57E-07 | 6.3397 | 4.12E-06 | 5.3854 |
| Myristic acid | 8.2377 | 3.0422 | 6.49E-07 | 6.1874 | 5.54E-06 | 5.2567 |
| L-Glutamic acid | 13.603 | 3.7659 | 7.06E-07 | 6.1513 | 5.72E-06 | 5.2428 |
| L-Isoleucine | 7.8235 | 2.9678 | 7.58E-07 | 6.1206 | 5.84E-06 | 5.2333 |
| Aminoadipic acid | 6.8707 | 2.7804 | 1.30E-06 | 5.887 | 9.55E-06 | 5.0199 |
| 2-Hydroxyethanesulfonate | 46.487 | 5.5388 | 1.40E-06 | 5.8524 | 9.89E-06 | 5.0046 |
| Stearic acid | 10.72 | 3.4222 | 2.00E-06 | 5.6999 | 1.35E-05 | 4.8706 |
| Threonic acid | 0.26021 | -1.9423 | 2.11E-06 | 5.6749 | 1.37E-05 | 4.8634 |
| Heptadecanoic acid | 8.4434 | 3.0778 | 2.48E-06 | 5.6059 | 1.54E-05 | 4.8113 |
| Erucic acid | 13.07 | 3.7082 | 1.51E-05 | 4.8215 | 9.05E-05 | 4.0434 |
| Carnitine | 1659.9 | 10.697 | 1.74E-05 | 4.7598 | 0.000101 | 3.9974 |
| Oxoglutaric acid | 11.276 | 3.4952 | 1.82E-05 | 4.7393 | 0.000102 | 3.9922 |
| 3-Methyl-2-oxovaleric acid | 8.7423 | 3.128 | 2.35E-05 | 4.6297 | 0.000127 | 3.8973 |
| Citric acid | 6.3385 | 2.6642 | 2.53E-05 | 4.596 | 0.000132 | 3.8779 |
| Serine | 3.3189 | 1.7307 | 3.18E-05 | 4.4973 | 0.000161 | 3.7929 |
| Ribothymidine | 5.4305 | 2.4411 | 3.56E-05 | 4.4482 | 0.000174 | 3.7595 |
| 2-Octenoic acid | 4.4371 | 2.1496 | 3.65E-05 | 4.4376 | 0.000174 | 3.7595 |
| cis-Aconitic acid | 6.2594 | 2.646 | 3.78E-05 | 4.4228 | 0.000175 | 3.7574 |
| Pantothenic acid | 5.6693 | 2.5032 | 3.97E-05 | 4.4016 | 0.000178 | 3.7484 |
| Galacturonic acid | 7.88 | 2.9782 | 4.10E-05 | 4.3877 | 0.000179 | 3.7464 |
| L-Tyrosine | 2.7958 | 1.4833 | 4.90E-05 | 4.3096 | 0.000209 | 3.6799 |
| L-Lysine | 3.3052 | 1.7247 | 5.94E-05 | 4.2259 | 0.000247 | 3.6075 |
| N6-Acetyl-L-lysine | 10.808 | 3.4341 | 6.15E-05 | 4.2114 | 0.000249 | 3.6039 |
| Pentadecanoic acid | 5.4132 | 2.4365 | 8.38E-05 | 4.0769 | 0.000331 | 3.4801 |
| Homocitrulline | 28.097 | 4.8123 | 9.66E-05 | 4.0151 | 0.000373 | 3.4289 |
| Trigonelline | 0.4546 | -1.1373 | 0.000105 | 3.9777 | 0.000397 | 3.4016 |
| Dimethylglycine | 12.52 | 3.6462 | 0.000118 | 3.9295 | 0.000433 | 3.3634 |
| Phenol | 5.2681 | 2.3973 | 0.000136 | 3.867 | 0.000489 | 3.3107 |
| Glycine | 5.8211 | 2.5413 | 0.000316 | 3.4999 | 0.001114 | 2.9532 |
| Creatinine | 11.701 | 3.5485 | 0.000341 | 3.4673 | 0.001175 | 2.9299 |
| N-Alpha-acetyllysine | 4.0166 | 2.006 | 0.000461 | 3.3359 | 0.001557 | 2.8077 |
| myo-Inositol | 4.6291 | 2.2107 | 0.000534 | 3.2724 | 0.001733 | 2.7611 |
| Threitol | 17.291 | 4.112 | 0.000535 | 3.2716 | 0.001733 | 2.7611 |
| Phosphoric acid | 2.9426 | 1.5571 | 0.000569 | 3.2447 | 0.001779 | 2.7498 |
| Methionine sulfoxide | 2.0595 | 1.0423 | 0.000571 | 3.2433 | 0.001779 | 2.7498 |
| Dodecanoic acid | 4.0573 | 2.0205 | 0.000589 | 3.2297 | 0.001801 | 2.7444 |
| 3-Methyl-2-oxovaleric acid | 7.4074 | 2.889 | 0.000613 | 3.2124 | 0.00184 | 2.7353 |
| L-Arginine | 3.4234 | 1.7754 | 0.000877 | 3.0568 | 0.002557 | 2.5922 |
| 5-Hydroxylysine | 3.0651 | 1.6159 | 0.000888 | 3.0516 | 0.002557 | 2.5922 |
| Sulfolithocholic acid | 0.40006 | -1.3217 | 0.001515 | 2.8197 | 0.00423 | 2.3736 |
| L-Asparagine | 3.4645 | 1.7926 | 0.001683 | 2.7739 | 0.004621 | 2.3352 |
| L-Allothreonine | 4.6182 | 2.2073 | 0.001775 | 2.7509 | 0.004792 | 2.3195 |
| Indoxyl sulfate | 0.45419 | -1.1386 | 0.002539 | 2.5953 | 0.006744 | 2.1711 |
| L-Glutamic acid | 8.1987 | 3.0354 | 0.002861 | 2.5435 | 0.007475 | 2.1264 |
| Citrulline | 3.2454 | 1.6984 | 0.003214 | 2.4929 | 0.008265 | 2.0827 |
| 3,4,5-Trimethoxycinnamic acid | 0.31904 | -1.6482 | 0.00603 | 2.2197 | 0.015029 | 1.8231 |
| Pipecolic acid | 6.0662 | 2.6008 | 0.006922 | 2.1598 | 0.016573 | 1.7806 |
| *13C10,15N5-AMP | 4.3505 | 2.1212 | 0.006938 | 2.1588 | 0.016573 | 1.7806 |
| D-threo-Isocitric acid | 6.5356 | 2.7083 | 0.007059 | 2.1513 | 0.016573 | 1.7806 |
| Pyrrolidonecarboxylic acid | 3.555 | 1.8299 | 0.007799 | 2.108 | 0.017697 | 1.7521 |
| Homogentisic acid | 2.2586 | 1.1754 | 0.007861 | 2.1045 | 0.017697 | 1.7521 |
| Glucuronic acid | 8.5979 | 3.104 | 0.007866 | 2.1043 | 0.017697 | 1.7521 |
| L-Alanine | 4.4679 | 2.1596 | 0.009172 | 2.0375 | 0.020355 | 1.6913 |
| Xylitol | 4.1348 | 2.0478 | 0.010507 | 1.9785 | 0.023002 | 1.6382 |
| 2-Isopropylmalic acid | 0.28824 | -1.7946 | 0.011181 | 1.9515 | 0.024151 | 1.6171 |
| *13C,15N-UMP | 5.0717 | 2.3425 | 0.015375 | 1.8132 | 0.032348 | 1.4902 |
| Orotidine | 6.9121 | 2.7891 | 0.018338 | 1.7366 | 0.037135 | 1.4302 |
| Sebacic acid | 2.6469 | 1.4043 | 0.029444 | 1.531 |  |  |
| L-Proline | 2.3305 | 1.2206 | 0.044177 | 1.3548 |  |  |
| Fructose | 0.48402 | -1.0469 | 0.047277 | 1.3253 |  |  |

**Supplementary table 2: Significant differences of metabolites in plasma collected from Established disease cohort – Participants diagnosed with a fetal growth restricted infant, compared to gestation matched controls.**

|  | FC | log2(FC) | P value | -LOG10(p) | Q Value | -LOG10(p) |
| --- | --- | --- | --- | --- | --- | --- |
| Levulinic acid | 524.79 | 9.0356 | 1.9957e-07 | 6.6999 | 1.8172e-05 | 4.7406 |
| Pentadecanoic acid | 697.13 | 9.4453 | 2.2435e-07 | 6.6491 | 1.8172e-05 | 4.7406 |
| L-Valine | 669.13 | 9.3861 | 3.9822e-06 | 5.3999 | 0.000215 | 3.6675 |
| L-Lysine | 33.637 | 5.072 | 5.9905e-06 | 5.2225 | 0.000243 | 3.6151 |
| 2,6-Dihydroxybenzoic acid | 26.964 | 4.7529 | 8.8361e-06 | 5.0537 | 0.000286 | 3.5432 |
| Xanthine | 9.2632 | 3.2115 | 2.8531e-05 | 4.5447 | 0.000676 | 3.17 |
| 1,3-Dimethyluric acid | 14.349 | 3.8428 | 3.3253e-05 | 4.4782 | 0.000676 | 3.17 |
| 1,5-Anhydrosorbitol | 14.367 | 3.8447 | 3.3386e-05 | 4.4764 | 0.000676 | 3.17 |
| Heptadecanoic acid | 23.736 | 4.569 | 4.3382e-05 | 4.3627 | 0.000781 | 3.1074 |
| Pyrrolidonecarboxylic acid | 10.878 | 3.4433 | 6.7135e-05 | 4.1731 | 0.001088 | 2.9635 |
| L-Aspartic acid | 549.35 | 9.1016 | 0.000164 | 3.785 | 0.00215 | 2.6675 |
| Ethylmalonic acid | 8.6691 | 3.1159 | 0.000176 | 3.7537 | 0.00215 | 2.6675 |
| Citric acid | 373.79 | 8.5461 | 0.000179 | 3.7475 | 0.00215 | 2.6675 |
| Alpha-Tocopherol | 25.044 | 4.6464 | 0.000186 | 3.7309 | 0.00215 | 2.6675 |
| L-Alanine | 20.045 | 4.3252 | 0.000227 | 3.6435 | 0.002454 | 2.6101 |
| Hypotaurine | 6.4349 | 2.6859 | 0.000265 | 3.5768 | 0.002683 | 2.5714 |
| Maltotriose | 5.3261 | 2.4131 | 0.000473 | 3.325 | 0.004282 | 2.3684 |
| N-Acetyl-L-alanine | 7.553 | 2.9171 | 0.000476 | 3.3226 | 0.004282 | 2.3684 |
| PA(16:0/16:0) | 5.7144 | 2.5146 | 0.000529 | 3.2769 | 0.004506 | 2.3462 |
| 7-Methylguanine | 762.09 | 9.5738 | 0.000572 | 3.2426 | 0.004634 | 2.3341 |
| N-Acetylneuraminic acid | 4.3064 | 2.1065 | 0.000725 | 3.1398 | 0.005434 | 2.2649 |
| Phenol | 2.857 | 1.5145 | 0.000738 | 3.132 | 0.005434 | 2.2649 |
| Undecanedioic acid | 3.9461 | 1.9804 | 0.001029 | 2.9875 | 0.007249 | 2.1397 |
| Isomaltose | 5.4736 | 2.4525 | 0.001278 | 2.8935 | 0.008626 | 2.0642 |
| Nutriacholic acid | 5.5751 | 2.479 | 0.001616 | 2.7915 | 0.010473 | 1.9799 |
| N-Acetylglutamine | 3.7753 | 1.9166 | 0.002171 | 2.6634 | 0.012984 | 1.8866 |
| 2-Hydroxyethanesulfonate | 7.2506 | 2.8581 | 0.002186 | 2.6603 | 0.012984 | 1.8866 |
| *13C,15N-UMP | 5.4175 | 2.4376 | 0.002244 | 2.649 | 0.012984 | 1.8866 |
| L-Arginine | 0.35605 | -1.4899 | 0.002872 | 2.5418 | 0.015509 | 1.8094 |
| Citrulline | 0.31177 | -1.6815 | 0.003001 | 2.5227 | 0.015682 | 1.8046 |
| Methylcysteine | 11.305 | 3.4989 | 0.003403 | 2.4681 | 0.016212 | 1.7902 |
| 3-Methylhistidine | 6.8685 | 2.78 | 0.003406 | 2.4678 | 0.016212 | 1.7902 |
| Palmitic acid | 5.6003 | 2.4855 | 0.00349 | 2.4572 | 0.016212 | 1.7902 |
| Trigonelline | 5.8064 | 2.5377 | 0.003503 | 2.4556 | 0.016212 | 1.7902 |
| Glyceric acid | 11.741 | 3.5535 | 0.004345 | 2.362 | 0.019551 | 1.7088 |
| L-Kynurenine | 10.31 | 3.366 | 0.004963 | 2.3042 | 0.021732 | 1.6629 |
| Indoxyl sulfate | 0.44097 | -1.1812 | 0.005656 | 2.2475 | 0.024111 | 1.6178 |
| Creatinine | 298.11 | 8.2197 | 0.006473 | 2.1889 | 0.026889 | 1.5704 |
| Linoleic acid | 7.7402 | 2.9524 | 0.006706 | 2.1736 | 0.027158 | 1.5661 |
| Behenic acid | 23.288 | 4.5415 | 0.007561 | 2.1214 | 0.029876 | 1.5247 |
| Galacturonic acid | 3.9042 | 1.965 | 0.007829 | 2.1063 | 0.030197 | 1.52 |
| Galactitol | 5.3164 | 2.4105 | 0.008486 | 2.0713 | 0.031969 | 1.4953 |
| Alpha-Linolenic acid | 3.8976 | 1.9626 | 0.00928 | 2.0325 | 0.034167 | 1.4664 |
| L-Cysteine | 2.6676 | 1.4155 | 0.011867 | 1.9256 | 0.042388 | 1.3728 |
| Uridine | 6.4608 | 2.6917 | 0.012062 | 1.9186 | 0.042388 | 1.3728 |
| Methionine sulfoxide | 3.3096 | 1.7267 | 0.014593 | 1.8359 | 0.049251 | 1.3076 |
| Nonadecanoic acid | 2.1946 | 1.134 | 0.017121 | 1.7665 | 0.056605 | 1.2471 |
| Sulfolithocholic acid | 2.9148 | 1.5434 | 0.018564 | 1.7313 | 0.060147 | 1.2208 |
| Methylsuccinic acid | 5.4931 | 2.4576 | 0.021423 | 1.6691 | 0.068048 | 1.1672 |
| 2-Isopropylmalic acid | 3.676 | 1.8781 | 0.025755 | 1.5891 | 0.078891 | 1.103 |
| Alpha-Hydroxyisobutyric acid | 3.733 | 1.9004 | 0.02581 | 1.5882 | 0.078891 | 1.103 |
| Homocitrulline | 0.4985 | -1.0043 | 0.02762 | 1.5588 | 0.08286 | 1.0817 |
| Fumaric acid | 6.4184 | 2.6822 | 0.032079 | 1.4938 | 0.091171 | 1.0401 |
| Xylitol | 2.6733 | 1.4186 | 0.037269 | 1.4287 |  |  |
| L-Isoleucine | 9.264 | 3.2116 | 0.037409 | 1.427 |  |  |
| Beta-N-Acetylglucosamine | 2.8131 | 1.4922 | 0.041517 | 1.3818 |  |  |
| cis-Aconitic acid | 3.7024 | 1.8885 | 0.041928 | 1.3775 |  |  |
| Alpha-D-Glucose | 2.0368 | 1.0263 | 0.043049 | 1.366 |  |  |
| Sebacic acid | 3.0674 | 1.617 | 0.043298 | 1.3635 |  |  |
| 3-Oxocholic acid | 4.4852 | 2.1652 | 0.043496 | 1.3615 |  |  |
| Pantothenic acid | 3.3972 | 1.7643 | 0.054143 | 1.2665 |  |  |
| Taurine | 3.5482 | 1.8271 | 0.0575 | 1.2403 |  |  |
| Oxalacetic acid | 2.9176 | 1.5448 | 0.065006 | 1.187 |  |  |
| L-Proline | 8.6575 | 3.114 | 0.072856 | 1.1375 |  |  |
| Hypoxanthine | 0.4709 | -1.0865 | 0.080993 | 1.0916 |  |  |
| PC(14:0/14:0) | 2.6484 | 1.4051 | 0.087907 | 1.056 |  |  |
| L-Cystine | 2.1476 | 1.1028 | 0.088165 | 1.0547 |  |  |
| Carnitine | 0.29134 | -1.7792 | 0.096774 | 1.0142 |  |  |

**Supplementary table 3: Significant differences of metabolites in plasma collected from BUMPS cohort – Participants later diagnosed with preeclampsia, compared to gestation matched controls.**

|  | FC | log2(FC) | raw.pval | -log10(p) |
| --- | --- | --- | --- | --- |
| Glutaric acid | 2.3531 | 1.2346 | 0.006835 | 2.1653 |
| 4-Aminopyridine | 0.41255 | -1.2774 | 0.008001 | 2.0969 |
| 2-Aminonicotinic acid | 0.42565 | -1.2323 | 0.008865 | 2.0523 |
| 3-Aminopropane-1-sulfonic acid | 0.40545 | -1.3024 | 0.011348 | 1.9451 |
| porphobilinogen | 3.5973 | 1.8469 | 0.015469 | 1.8105 |
| 4-Dodecylbenzenesulfonic acid | 0.46643 | -1.1003 | 0.016634 | 1.779 |
| 4-Undecylbenzenesulfonic acid | 0.46668 | -1.0995 | 0.021179 | 1.6741 |
| Aminohippuric acid | 2.4157 | 1.2724 | 0.022282 | 1.6521 |
| 1,5-Anhydro-6-deoxy-D-threo-hex-1-en-3-ulose | 2.1191 | 1.0834 | 0.023873 | 1.6221 |
| 3,4-dihydroxybutyric acid | 2.0909 | 1.0641 | 0.02486 | 1.6045 |
| irdabisant | 0.47255 | -1.0815 | 0.025668 | 1.5906 |
| (2R)-3-{[(2-Aminoethoxy)(hydroxy)phosphoryl]oxy}-2-[(1Z,11Z)-1,11-octadecadien-1-yloxy]propyl (6Z,9Z,12Z,15Z)-6,9,12,15-octadecatetraenoate | 2.3754 | 1.2482 | 0.03279 | 1.4843 |
| 4-Methyleneglutamic acid | 2.867 | 1.5195 | 0.040843 | 1.3889 |
| Methylsuccinic acid | 3.4035 | 1.767 | 0.043325 | 1.3633 |
| D-(?)-Erythrulose | 2.0228 | 1.0163 | 0.04367 | 1.3598 |
| 1-(1Z-hexadecenyl)-2-oleoyl-sn-glycero-3-phosphoethanolamine | 2.5564 | 1.3541 | 0.061024 | 1.2145 |
| 4-Acetamidobutanoic acid | 3.002 | 1.5859 | 0.063081 | 1.2001 |
| (1R)-6-Hydroxy-7-methoxy-1-methyl-1,2,3,4-tetrahydro-1-isoquinoliniumcarboxylate | 2.3631 | 1.2407 | 0.065537 | 1.1835 |
| 1-(1Z-octadecenyl)-2-linoleoyl-sn-glycero-3-phosphoethanolamine | 2.4908 | 1.3166 | 0.071017 | 1.1486 |
| 5-[(3Z)-5-Hydroxy-3-methyl-3-penten-1-yl]-1,4a-dimethyl-6-methylenedecahydro-1-naphthalenecarboxylic acid | 2.2796 | 1.1888 | 0.071184 | 1.1476 |
| 1-(1Z-octadecenyl)-2-(9Z-octadecenoyl)-sn-glycero-3-phosphoethanolamine | 2.1672 | 1.1159 | 0.072428 | 1.1401 |
| 1-(1Z-octadecenyl)-2-(4Z,7Z,10Z,13Z,16Z,19Z-docosahexaenoyl)-sn-glycero-3-phosphoethanolamine | 3.4929 | 1.8044 | 0.072664 | 1.1387 |
| 4-{[(3-Hydroxy-3-methylbutanoyl)oxy]methyl}-1-[(3-methylbutanoyl)oxy]-6,7a-dihydro-1H-spiro[cyclopenta[c]pyran-7,2'-oxiran]-6-yl 3-methylpentanoate | 2.4227 | 1.2766 | 0.0767 | 1.1152 |
| Acamprosate | 2.1155 | 1.081 | 0.077136 | 1.1127 |
| (2R)-3-{[(2-Aminoethoxy)(hydroxy)phosphoryl]oxy}-2-[(1Z,11Z)-1,11-octadecadien-1-yloxy]propyl (5Z,8Z,11Z,14Z)-5,8,11,14-icosatetraenoate | 3.5298 | 1.8196 | 0.078059 | 1.1076 |
| 1-(1Z-hexadecenyl)-2-(4Z,7Z,10Z,13Z,16Z,19Z-docosahexaenoyl)-sn-glycero-3-phosphoethanolamine | 2.66 | 1.4114 | 0.086767 | 1.0616 |
| a-Hydroxyhippuric acid | 2.0333 | 1.0238 | 0.087135 | 1.0598 |
| DEHYDROASCORBIC ACID | 0.44999 | -1.152 | 0.088673 | 1.0522 |
| Oxetacaine | 3.0608 | 1.6139 | 0.09659 | 1.0151 |
| Cholesterol sulfate | 3.7419 | 1.9038 | 0.098339 | 1.0073 |

**Supplementary table 4: Significant differences of metabolites in plasma collected from BUMPS cohort – Participants later diagnosed with a fetal growth restricted infant, compared to gestation matched controls.**

|  | FC | log2(FC) | raw.pval | -log10(p) |
| --- | --- | --- | --- | --- |
| (2R)-3-sulfopropanediol | 0.24822 | -2.0103 | 0.010624 | 1.9737 |
| 3-Aminopropane-1-sulfonic acid | 0.4763 | -1.07 | 0.010624 | 1.9737 |
| Glutaric acid | 2.0092 | 1.0066 | 0.062995 | 1.2007 |
| 1-Deoxy-L-mannitol | 2.6238 | 1.3916 | 0.082233 | 1.085 |
| 3-Methoxybenzenepropanoic acid | 2.4115 | 1.2699 | 0.082296 | 1.0846 |

**Supplementary table 5: Top 10 univariate Receiver operating characteristics (ROC) curves for metabolites across established and BUMPS cohort. AUC = Area under the curve**

|  | AUC | Pval |
| --- | --- | --- |
| **Established Control and Preeclampsia** | | |
| L-Cystine | 0.9568 | 1.79E-16 |
| L-Cysteine | 0.9536 | 1.01E-15 |
| 3'-Sialyllactose | 0.9384 | 4.47E-12 |
| Alpha-Hydroxyisobutyric acid | 0.9224 | 4.38E-11 |
| L-Methionine | 0.908 | 5.00E-12 |
| 3-Hydroxybutyric acid | 0.8912 | 6.18E-08 |
| L-Acetylcarnitine | 0.8824 | 1.57E-08 |
| L-Leucine | 0.8824 | 4.61E-10 |
| Cortisol | 0.8816 | 3.62E-08 |
| L-Phenylalanine | 0.8776 | 1.23E-09 |
| **Established Control and Fetal growth restriction** | | |
| Pentadecanoic acid | 0.92 | 2.24E-07 |
| Levulinic acid | 0.908571 | 2.00E-07 |
| L-Valine | 0.9 | 3.98E-06 |
| L-Lysine | 0.882857 | 5.99E-06 |
| 2,6-Dihydroxybenzoic acid | 0.865714 | 8.84E-06 |
| Heptadecanoic acid | 0.857143 | 4.34E-05 |
| 1,3-Dimethyluric acid | 0.851429 | 3.33E-05 |
| 1,5-Anhydrosorbitol | 0.851429 | 3.34E-05 |
| Phenol | 0.851429 | 0.000738 |
| Xanthine | 0.848571 | 2.85E-05 |
| **BUMPS Control and Preeclampsia** | | |
| (5-Oxo-2-sulfo-2,5-dihydro-2-furanyl)acetic acid | 0.676816 | 0.004832 |
| porphobilinogen | 0.670406 | 0.015469 |
| Pyrogallol-2-O-glucuronide | 0.668269 | 0.023234 |
| Glutaric acid | 0.665598 | 0.006835 |
| 4-Dodecylbenzenesulfonic acid | 0.663462 | 0.016634 |
| 2-Furoylglycine | 0.662927 | 0.032139 |
| 2-Aminonicotinic acid | 0.656517 | 0.008865 |
| oxiracetam | 0.654915 | 0.020365 |
| irdabisant | 0.653846 | 0.025668 |
| Dodecyl sulfate | 0.652778 | 0.030955 |
| **BUMPS Control and Fetal growth restriction** | | |
| 3-Aminopropane-1-sulfonic acid | 0.666063 | 0.010624 |
| 3-Methylhistidine | 0.641606 | 0.014152 |
| Pyrogallol-2-O-sulphate | 0.641304 | 0.006889 |
| (4S,9R,11R)-9,11-Dihydroxy-29-(4-hydroxyphenyl)-4-methyl-3-nonacosanone | 0.62651 | 0.058361 |
| Nitrosoguvacoline | 0.622283 | 0.053743 |
| N-Tridecanoylglycine | 0.621075 | 0.023636 |
| 4-Acetamidobutanoic acid | 0.619565 | 0.171097 |
| Urocanic acid | 0.616244 | 0.020895 |
| Glutaric acid | 0.613829 | 0.062995 |
| (3R,4S,9R,11R)-27-(4-Hydroxyphenyl)-4-methyl-3,9,11-heptacosanetriol | 0.612017 | 0.105401 |
